# p53 Plays a Role in Mesenchymal Differentiation Programs, in a Cell Fate Dependent Manner

**DOI:** 10.1371/journal.pone.0003707

**Published:** 2008-11-12

**Authors:** Alina Molchadsky, Igor Shats, Naomi Goldfinger, Meirav Pevsner-Fischer, Melissa Olson, Ariel Rinon, Eldad Tzahor, Guillermina Lozano, Dov Zipori, Rachel Sarig, Varda Rotter

**Affiliations:** 1 Department of Molecular Cell Biology, Weizmann Institute of Science, Rehovot, Israel; 2 Department of Cancer Genetics, The University of Texas M. D. Anderson Cancer Center, Houston, Texas, United States of America; 3 Department of Biological Regulation, Weizmann Institute of Science, Rehovot, Israel; Victor Chang Cardiac Research Institute, Australia

## Abstract

**Background:**

The tumor suppressor p53 is an important regulator that controls various cellular networks, including cell differentiation. Interestingly, some studies suggest that p53 facilitates cell differentiation, whereas others claim that it suppresses differentiation. Therefore, it is critical to evaluate whether this inconsistency represents an authentic differential p53 activity manifested in the various differentiation programs.

**Methodology/Principal Findings:**

To clarify this important issue, we conducted a comparative study of several mesenchymal differentiation programs. The effects of p53 knockdown or enhanced activity were analyzed in mouse and human mesenchymal cells, representing various stages of several differentiation programs. We found that p53 down-regulated the expression of master differentiation-inducing transcription factors, thereby inhibiting osteogenic, adipogenic and smooth muscle differentiation of multiple mesenchymal cell types. In contrast, p53 is essential for skeletal muscle differentiation and osteogenic re-programming of skeletal muscle committed cells.

**Conclusions:**

These comparative studies suggest that, depending on the specific cell type and the specific differentiation program, p53 may exert a positive or a negative effect, and thus can be referred as a “guardian of differentiation” at large.

## Introduction

The tumor suppressor p53 known as the “guardian of the genome”, plays a key role in prevention of cancer development [1]. p53 is a sequence specific transcription factor that binds to DNA and thereby activates specific transcription of target genes [2], [3] such as p21 [4]. Activation of p53 induces cellular programs including cell cycle arrest, apoptosis or senescence which prevent accumulation of genetically altered cells [5], [6]. Moreover, p53 was found to play a role in angiogenesis, invasion and motility, glycolysis and autophagy [7], [8], [9], [10], [11]. These findings suggest that p53 acts as an important general regulator that controls various cellular gene networks [12].

Although the tumor suppressing activity of p53 has been clearly established, less attention was given to its possible involvement in the processes of cell differentiation and development. During embryogenesis the transcription of p53 is tightly regulated (reviewed in [13], yet initial analysis of p53 null mice failed to detect any apparent developmental defects [14]. However, further detailed studies revealed several abnormalities in at least some p53 null embryos. These included upper incisor fusion, ocular abnormalities, polydactyly of the hindlimbs and exencephaly that was exhibited by female embryos leading to a fatal phenotype [15]. p53 null adult males and females show reduced fertility due to impaired spermatogenesis or embryonic implantation, respectively [16], [17], [18]. In contrast to the apparent normal development of most p53 null mice, with only sporadic developmental defects, severe gastrulation defects have been observed in p53 deficient Xenopus embryos [19]. One possible explanation for this discrepancy could be the fact that in the mouse, the p53 family members p63 and p73, which are expressed in early mouse embryos may compensate for the loss of p53, resulting in the incomplete penetrance of the developmental phenotype observed, whereas in frogs p53 is solely responsible for early embryogenesis [11]. In addition to the major role of p53 during Xenopus development, recent studies in zebrafish demonstrate its function in the control of proper gut and neuronal development [20], [21]. Inhibiting p53 in the salamander prevents limb regeneration [22]. These findings demonstrate the essential role of p53 during various differentiation processes of divergent organisms. The discovery that lack of p53 in the mouse can lead to a fatal defect, clearly implicates p53 as an important factor in development. The natural selection of developmentally normal p53 null mice, in which compensation by other p53 family members most likely masks possible significant functions that p53 may play during differentiation processes, and the influence of the specific mouse strain on the ratio of affected mice [13], indicate the complexity associated with this *in vivo* model.

*In vitro* models and recent advances in gene knockdown techniques enabled focusing on the specific function of p53 during various differentiation programs. Indeed, a growing body of data derived from *in vitro* models suggests that p53 plays a major regulatory role in cell differentiation. Interestingly, however, it was noticed that under some circumstances p53 facilitates cell differentiation whereas in others, it seems to be suppressive [23], [24]. For example, introduction of wild-type (wt) p53 into undifferentiated early pre-B cell line, which lacks p53 induced their differentiation [25], [26]. In addition, irradiation of another pre-B cell line containing wt p53 induced differentiation, which was inhibited by mutant p53, suggesting that radiation-induced p53 activation was the differentiation inducer [27]. Induction towards differentiation by addition of exogenous wt p53 and block of normal differentiation pathways by addition of dominant negative mutant versions of p53 was observed in several other cell types as well, implying a positive role of p53 in these systems. The latter include myoblasts, keratinocytes, oligodendrocytes, neurons and thyroid cells [28]. However, recent studies demonstrated a multifaceted function of p53 during specific differentiation programs. These include mainly its involvement in osteogenic differentiation. Hüttinger-Kirchhof et al. [29], showed that p53 deficiency leads to inhibition of osteogenesis, while others demonstrated that it functions as a negative regulator of osteoblast differentiation, skeletal development and bone remodeling [30], [31], [32]. A major difference between these studies is the use of cell systems, which imply a diverse role for p53 in a cell type dependent manner. Similarly, while p53 was shown to be required for differentiation of skeletal myogenic cells [33], [34], [35], a recent study proposed that it is required for the tumor necrosis factor alpha-mediated inhibition of myogenesis [36]. During adipogenic differentiation of 3T3-L1 cells, p53 was shown to be downregulated and to exhibit a reduction in its DNA binding activity, suggesting its role as a negative regulator of adipogenesis [37], [38]. p53 may also inhibit myofibroblasts differentiation, since its inactivation in cancer associated fibroblasts is predicted to contribute to their myofibroblast phenotype [39]. Notably, despite extensive evidence regarding the involvement of p53 in diverse differentiation processes, its role in myofibroblast and adipocyte differentiation is still largely unknown.

Recent findings suggesting an inhibitory role for p53 in several differentiation systems, which challenge the previous dogma, claiming that p53 facilitates differentiation, prompted us to re-evaluate its role in several well characterized *in vitro* cell differentiation models. We have conducted a comparative study of mouse and human mesenchymal cells, representing various differentiation programs and evaluated the effect of either p53 knock-down or enhanced activity on their capacity to undergo a specific cell differentiation program. Our data demonstrate that p53 inhibits osteogenic, adipogenic and myofibroblast differentiation of mesenchymal progenitor cells, while it is essential for skeletal muscle differentiation and osteogenic re-programming of skeletal muscle committed cells. At the molecular level, p53 downregulates the expression of the master differentiation-inducing transcription factors myocardin (Myocd), Peroxisome prolifirator-activated receptor γ (PPARγ) , and osterix. These results disclose a new function of p53 during the differentiation of adipocytes and myofibroblasts and further stress the versatile nature of p53 function along various differentiation programs, suggesting that its function is dependent on the context of the specific cell fate. Our novel findings regarding p53 regulatory function during adipogenesis suggest its possible influence on fatty acid metabolism, which may affect energy production, obesity and membrane synthesis.

## Results

### Mouse embryonic fibroblasts derived from p53 knockout mice express elevated levels of defined groups of differentiation markers

**Figure 1:**
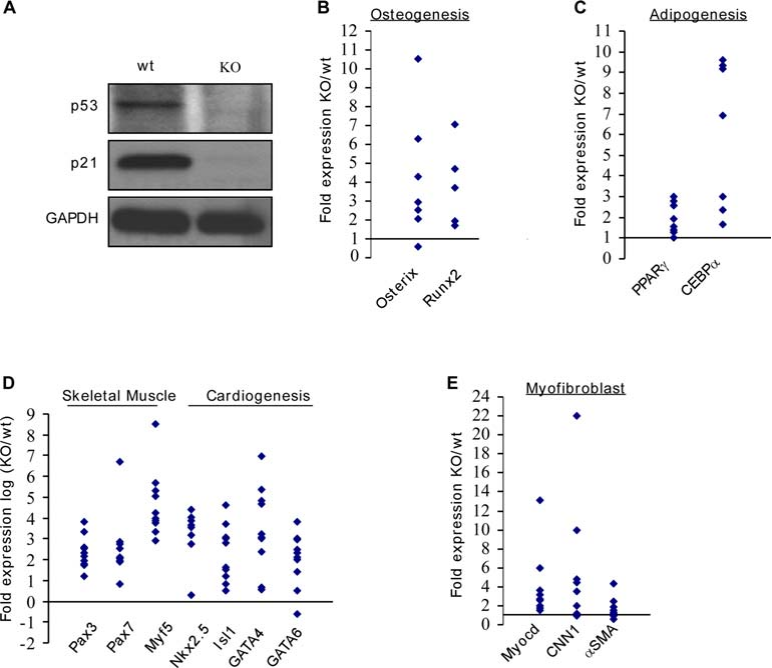
MEFs derived from p53 KO mice exhibit elevated levels of various differentiation markers. (A) p53 wt and p53 KO MEFs were subjected to Western blot analysis for p53 and p21. GAPDH serves as loading control. (B–E) Several isolations of MEFs derived from p53 KO or p53 wt mice were grown in culture for several passages. Relative basal expression levels of osteogenic differentiation markers (B); adipogenic differentiation markers (C); skeletal muscle and cardiogenic differentiation markers (D); and myofibroblast differentiation markers (E) were determined by QRT-PCR analysis. The scale in (D) is logarithmic. Dots indicate fold expression in KO relative to wt MEFs. Each dot represents the comparison of single MEFs pairs. The results are presented as a mean of two duplicate runs (N = 2) after normalization to HPRT control. Statistical significance of the differences between wt and KO MEFs was evaluated by paired one tailed Wilcoxon signed rank test and the p values for the genes are as follows: osterix p<0.023; Runx2 p<0.031; PPARγ p<0.004; CEBPα p<0.008; Pax3 p<0.001; Pax7 p<0.001; Myf5 p<0.001; Nkx2.5 p<0.001; Isl1 p<0.001; GATA4 p<0.001; GATA6 p<0.029; Myocd p<0.002; CNN1 p<0.012; αSMA p<0.039.

In order to obtain a general unbiased view of potential p53 involvement in various mesenchymal differentiation programs we compared the expression of diverse differentiation markers in wild-type (wt) mouse embryonic fibroblasts (MEFs) to those expressed by p53 knockout (KO) MEFs. MEFs obtained from 13.5 *dpc* embryos represent a heterogeneous population of primary adherent cells with variable differentiation capacity. Due to the potential heterogeneity of various MEFs preparations, we used several independent isolate of MEFs derived from wt and p53 KO embryos for quantitative real time RT-PCR (QRT-PCR) analysis. We found that absolute expression levels of diverse differentiation markers varied greatly between different MEF populations as well as between different passages of the same culture. Therefore, for the comparative analysis we used wt and p53 KO MEFs that were obtained from sibling embryos and cultured for identical periods. The absence of p53 expression and the concomitant downregulation of p21 in the p53 KO MEFs studied were verified by Western-blotting (Fig 1A). p53 KO MEFs consistently expressed higher levels of several mesenchymal differentiation markers. These included osterix and Runx2 of the osteogenic pathway (Fig 1B), the key adipogenic transcription factors PPARγ and CCAAT/enhancer binding protein α (CEBPα) (Fig 1C), early markers of skeletal muscle differentiation and cardiac transcription factors (Fig 1D). The expression of Myocd, an important myofibroblast/smooth muscle transcription factor, as well as that of smooth muscle structural components, calponin (CNN1) and α-smooth muscle actin (αSMA) were also higher in p53 KO MEFs (Fig 1E). Collectively, these data revealed an intriguing picture of a general upregulation of diverse mesenchymal differentiation markers in p53 KO MEFs, which suggest an inhibitory role for p53 in these differentiation pathways. Therefore, we decided to test this hypothesis by focusing on defined stages of these differentiation programs.

### p53 inhibits osteogenic differentiation of mouse mesenchymal progenitor cells

**Figure 2:**
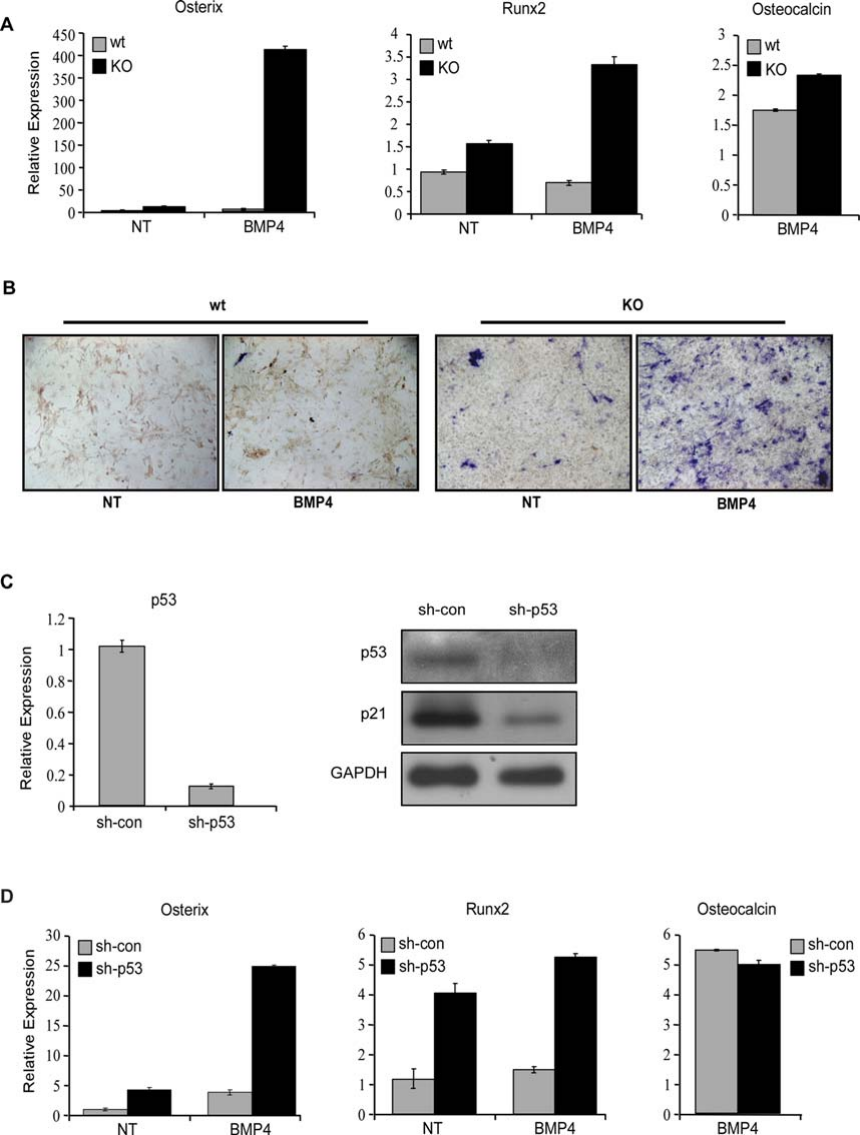
p53 inhibits MEFs differentiation towards the osteogenic lineage. (A) p53 wt and p53 KO MEFs were grown in culture for 48 h, then medium was changed to condition medium from 293T cells overexpressing BMP4 protein or to condition medium from control cells (NT). Total RNA was isolated 48 hours later. Using QRT-PCR analysis, relative expression of osterix, Runx2 and osteocalcin were determined following BMP4 treatement. (B) Alkaline Phosphatase activity in the experiment described in (A) was measured 4 days following BMP4 treatment. (C–D) p53 wt MEFs were infected with retrovirus encoding for either p53 directed sh-RNA (sh-p53) or control sh-RNA (sh-con). The relative expression level of p53 was determined by QRT-PCR (C). Western blot analysis was performed for p53 and p21. GAPDH serves as a loading control. (D) A similar experiment as (A) was performed in sh-p53 and sh-con MEFs. The results of QRT-PCR are presented as a range of two duplicate runs (N = 2) after normalization to HPRT control.

Previous data demonstrated a major regulatory role of p53 in osteogenic differentiation *in vitro* and in skeletal development and bone remodeling, *in vivo* [30], [31]. These studies demonstrated a negative regulatory role of p53 on two key osteogenic transcription factors, Runx2 and osterix. In agreement with these results, we observed elevated basal levels of these markers in p53 KO MEFs (Fig 1B), which prompted us to investigate the role of p53 during induction of this differentiation program. To this end, p53 KO MEFs and their wt counterparts were treated with bone morphogenetic protein 4 (BMP4), a known inducer of osteogenic differentiation, and analyzed by QRT-PCR. As can be seen in Figure 2A, the early osteogenic transcription factors, osterix and Runx2 were induced to significantly higher levels in p53 KO MEFs whereas induced levels of the terminal differentiation marker, The levels of osteocalcin, a late osteogenic differentiation marker were comparable in both MEFs types following BMP4 treatment. p53 KO MEFs exhibit also higher basal activity of the osteogenic marker alkaline-phosphatase (ALP) compared to wt MEFs. This enzymatic activity was further induced by BMP4 (Figure 2B), demonstrating that lack of p53 results in elevated activity of proteins involved in osteogenesis.

**Figure 3:**
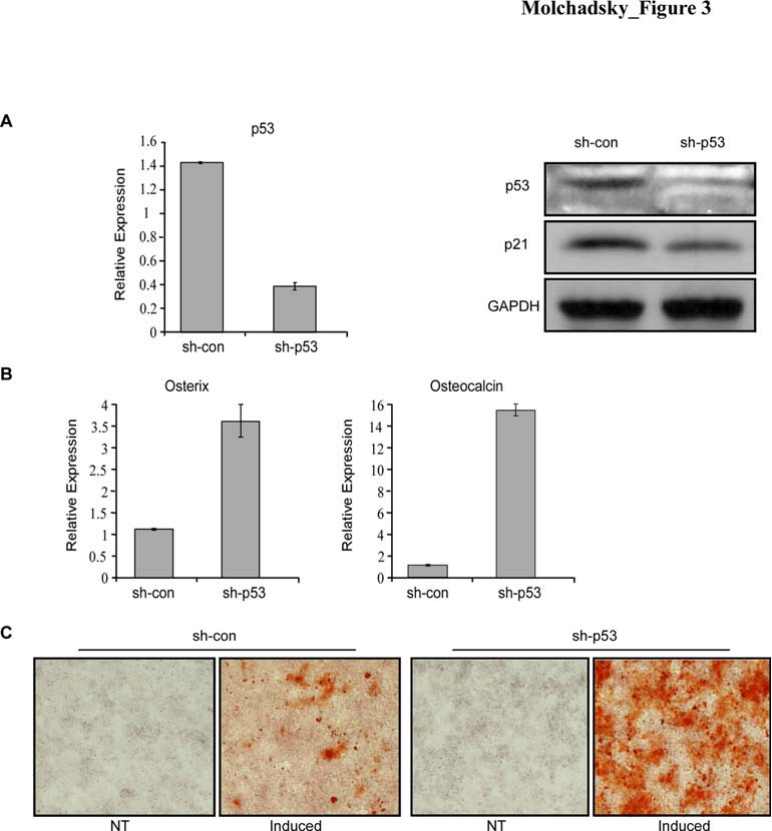
p53 inhibits osteogenic differentiation in MBA-15 cells. (A) MBA-15 cells were infected with retroviruses encoding for either p53 directed sh-RNA (sh-p53) or control sh-RNA (sh-con). The relative expression level of p53 was determined by QRT-PCR (left panel). Western blot analysis was performed for p53 and p21. GAPDH serves as a loading control (right panel). (B) Basal RNA expression levels for osterix and osteocalcin were determined by QRT-PCR. The results of QRT-PCR are presented as a range of two duplicate runs (N = 2) after normalization to HPRT control. (C) MBA-15-sh-p53 and MBA-15-sh-con cells were subjected to induction of osteogenic differentiation by treatment with medium containing 50 µg/ml L-ascorbic acid-2 phosphate, 10 Mm glycerol 2-phospate disodium salt and 10^−8^M dexamethasone (Induced) or with control medium (NT) for 6 days. Osteogenic differentiation was detected by Alizarin Red staining for the detection of Ca^2+^ precipitates.

As MEFs represent a heterogeneous population enriched with variable levels of multipotent precursors, it is important to determine whether the amplification observed is not a mere reflection of an increase in the number of such precursors in the p53 KO compared with the wt MEFs, or whether the cells exhibit an authentic higher levels of differentiation markers and increased differentiation capacity per cell. To this end we have knocked-down the endogenous p53 in a given individual wt MEFs population by infecting them with a retrovirus encoding for p53-directed small hairpin RNA (sh-p53). p53 inactivation was confirmed by both QRT-PCR and Western blot analysis (Fig 2C). The basal and induced expression levels of osteogenic differentiation markers of the MEFs pairs (sh-p53 and sh-control) were measured. As shown in Fig 2D, both basal and induced levels of osterix and Runx2 were upregulated in sh-p53 MEFs, while the induced level of osteocalcin was comparable in these MEF pairs. ALP activity was also elevated in sh-p53 compared to control MEFs (not shown). Thus, inhibiting p53 in a given MEF population, also results in increased levels of osteogenic differentiation markers, similarly to p53 KO MEFs, which indicate that p53 plays a specific regulatory role in osteogenic differentiation program. Since we could not induce terminal differentiation of MEFs in culture to typical osteoblasts, which give rise to Ca^2+^ precipitates, we have used the multipotent bone marrow stromal cell line, MBA-15, which can be induced to give rise to terminally differentiated osteoblasts, and represent a clonal, homogenous cell population [40]. First, we have knocked-down the expression of p53 in these cells, by sh-RNA (Fig 3A). Inhibiting the expression of p53 in MBA-15 cells resulted in elevated basal levels of both osterix and osteocalcin (Fig 3B). Induction towards osteogenic differentiation resulted in prominent Ca^2+^ precipitate formation in the MBA-15 sh-p53 cells compared to their controls, which exhibited sparse precipitate formation (Fig. 3C). These results, demonstrating a negative regulatory role of p53 on terminal differentiation of bone marrow stromal cells further support previous data showing a negative regulation of p53 during bone formation, *in vivo* by using an *in vitro* model [30], [31]. Thus, p53 negatively regulates key osteogenic transcription factors, resulting in restrained osteogenic differentiation of both MEFs and bone-marrow stromal cells, which correspond to two differentiation stages; while MEFs represent an early stage, reflecting the process of embryonic development, bone-marrow stromal cells are adult progenitor cells, which maintain proper bone differentiation and homeostasis.

### p53 inhibits the adipogenic differentiation program

**Figure 4:**
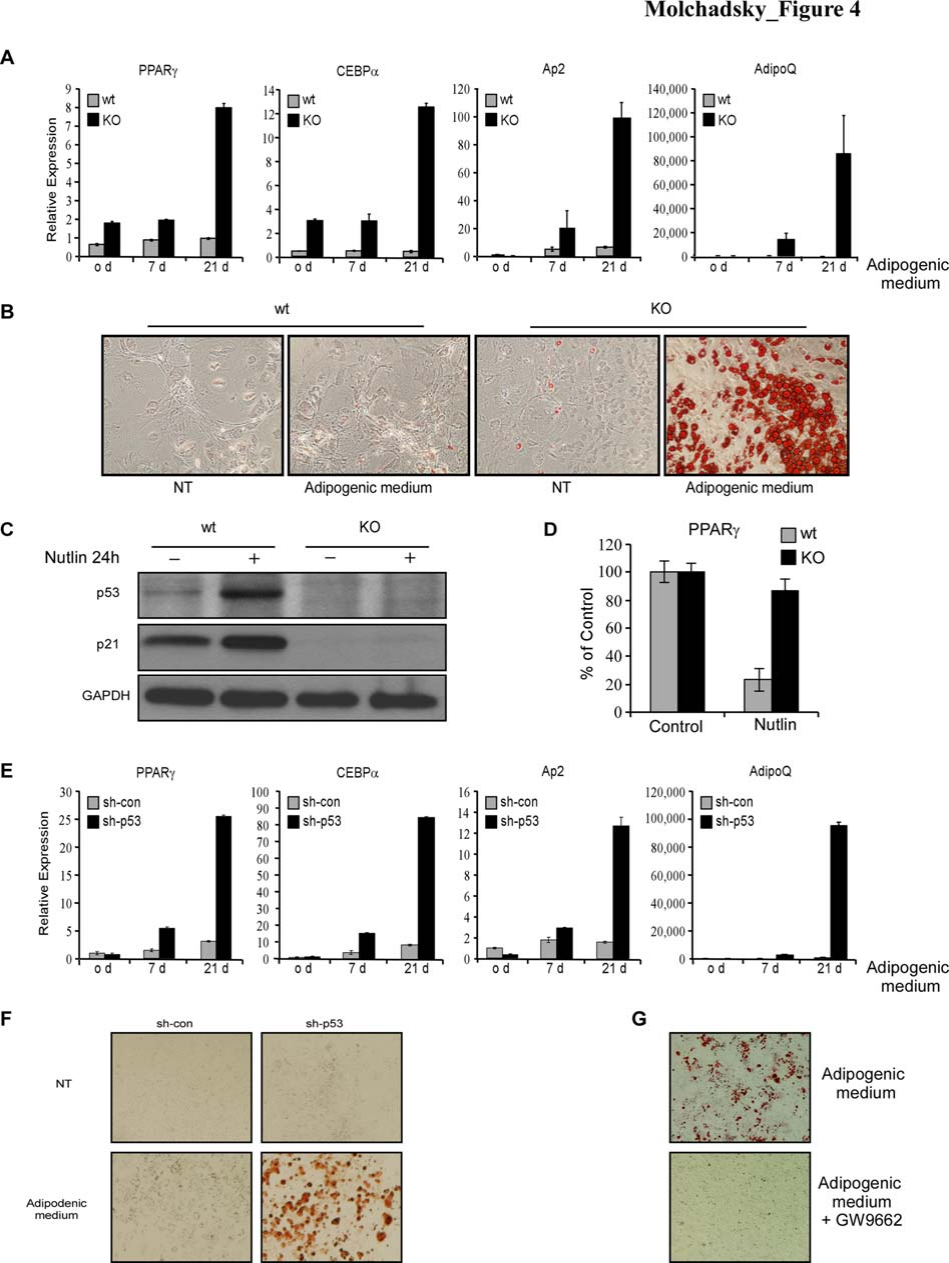
p53 inhibits the adipogenic differentiation program in MEFs. (A–B) Confluent cultures of p53 wt and p53 KO MEFs subjected to induction of adipogenic differentiation by treatment with medium containing 10 µg/ml insulin, 10^−6^M dexamethasone, 0.5 mM 3-Isobutyl-1-methylxanthine (Adipogenic medium) or with control medium (NT). The cells were grown for three weeks with medium replacement once in three days. Total RNA was isolated before the induction of differentiation (0d) as well as 7 and 21 days later. Relative expression of PPARγ, CEBPα, Ap2 and AdipoQ were determined by QRT-PCR analysis (A). Adipogenic differentiation was assessed using Oil Red O staining for lipid droplets (B). (C–D) p53 wt and p53 KO MEFs were grown in culture for 48 hours, then treated with Nutlin-3 at a final concentration of 25 µM, for 24 hours (Nutlin) or left untreated (Control). Western blot analysis was performed for p53 and p21.GAPDH serves as a loading control (C). Relative expression of PPARγ was determined by QRT-PCR. Normalized expression levels in control samples were set to 100% (D). (E–F) A similar experiment as A–B was performed in sh-p53 and sh-con MEFs. The results of QRT-PCR are presented as a range of two duplicate runs (N = 2) after normalization to HPRT control. (G) MEFs-sh-p53 cells were induced with adiogenic medium either in the presence of the PPARγ inhibitor GW9662, or without it. Adipogenic differentiation was assessed using Oil Red O staining for lipid droplets.

Our QRT-PCR analysis of multiple key differentiation markers in the MEFs pairs demonstrate, for the first time, elevated expression of the key adipogenic transcription factors PPARγ and CEBPα in p53 KO MEFs (Fig 1C). This suggests a negative regulation of p53 during adipogenesis, which may implicate a physiological role of p53 in fat metabolism. Therefore, we next aimed at evaluating the role of p53 in adipogenesis. The adipogenesis of MEFs by hormonal induction is a well-established model system for the study of adipocyte differentiation *in vitro* [41]. In order to examine the potential of p53 KO and wt MEFs to undergo adipogenic differentiation, these cells were treated with insulin and dexamethasone, and subjected to QRT-PCR analysis of various key adipogenic differentiation markers, at several time points. PPARγ is a transcription factor from the nuclear-receptor superfamily that is both necessary and sufficient for adipogenesis [42]. Consistent with its key role in adipocyte differentiation, the majority of known adipogenic regulators function through control of PPARγ expression or activity. CEBPα is an additional important factor in adipose tissue development [42]. Adipocyte fatty-acid-binding protein, AP-2 (FABP4) and Adiponectin (AdipoQ) are markers of differentiated adipocytes. Figure 4A demonstrates that basal and induced levels of PPARγ and CEBPα were significantly higher in p53 KO MEFs. The levels of AP-2 and AdipoQ markedly increased after induction. Importantly, Oil Red O staining for typical lipid droplets revealed a spontaneous adipocyte formation, even without hormonal induction, in p53 KO MEFs. Induction towards adipogenesis resulted in extensive adipocyte differentiation of p53 KO but not of wt MEFs (Fig 4B).

In order to test whether lower levels of adipogenic differentiation markers in wt MEFs represent a direct consequence of repression by p53, p53 KO and wt MEFs were treated with Nutlin-3, a small molecule inhibitor of the murine double minute (MDM2) gene [43]. Treatment of cells with this drug resulted in accumulation of p53 and activation of its downregulation target, p21 (Fig 4C). As demonstrated in Figure 4D, Nutlin-3 treatment resulted in a p53-dependent downregulation of PPARγ.

To verify that the effect of p53 deficiency on adipogenic differentiation is not a result of the heterogeneous nature of the MEFs population, we repeated these experiments using the sh-p53 and control MEFs described above. Similarly to p53 KO MEFs, the sh-p53 MEFs exhibited enhanced levels of the key adipogenic markers, and extensive mature adipocyte formation upon induction (Fig 4E, F).

We have repeated these experiments, using the multipotent MBA-15 cell line. While basal levels of PPARγ and AP-2 were not changed in sh-p53 MBA-15, compared to their control counterparts (the levels of CEBPα were even lower in sh-p53 cells), induced levels of PPARγ, adiponectin and AP-2 were elevated in sh-p53 MBA-15 compared to control cells (Suppl. Fig. S1a). Moreover, when these cells were induced to differentiate, prominent adipocyte colonies appeared only in the sh-p53 cells (Suppl. Fig. S1b).

As mentioned above, PPARγ is a major adipogenic factor. Activation of this nuclear receptor by ligands results in transcriptional activation of numerous factors participating in adipogenesis. Therefore, it is plausible to assume that p53 may regulate also downstream molecules of the adipogenic program, and exert its inhibitory effect not only through the regulation of PPARγ but through other downstream effectors as well. Indeed, as demonstrated in Figure 4A, the levels of CEBPα were higher in sh-p53 MEFs, both at basal and induced conditions. To determine whether facilitation of adipogenic differentiation in the absence of p53 is PPARγ-dependent, we used the irreversible PPARγ antagonist, GW9662. This compound was shown to prevent PPARγ activation by covalently modifying a cysteine residue in its ligand binding site. [44]. As shown in Figure 4G, addition of GW9662 to the induction medium of sh-p53 MEFs resulted in complete inhibition of adipocyte differentiation, suggesting that upregulation of other important adipogenic transcription factors such as CEBPα, in p53-deficient cells cannot compensate PPARγ inhibition.

Taken together, our results demonstrate that p53 inhibits adipogenic differentiation in two mesenchymal stem cells populations, representing different stages, as mentioned above. Given the established key role of PPARγ and CEBPα transcription factors in adipogenesis, their transcriptional repression by p53 is likely to contribute to the observed inhibitory effect of p53 on adipogenic differentiation. Of note, these results imply a regulatory role of p53 in the process of fat accumulation, consequently controlling the major metabolic energy source.

### p53 negatively regulates myofibroblast/smooth muscle differentiation by inhibiting the expression of Myocd

**Figure 5:**
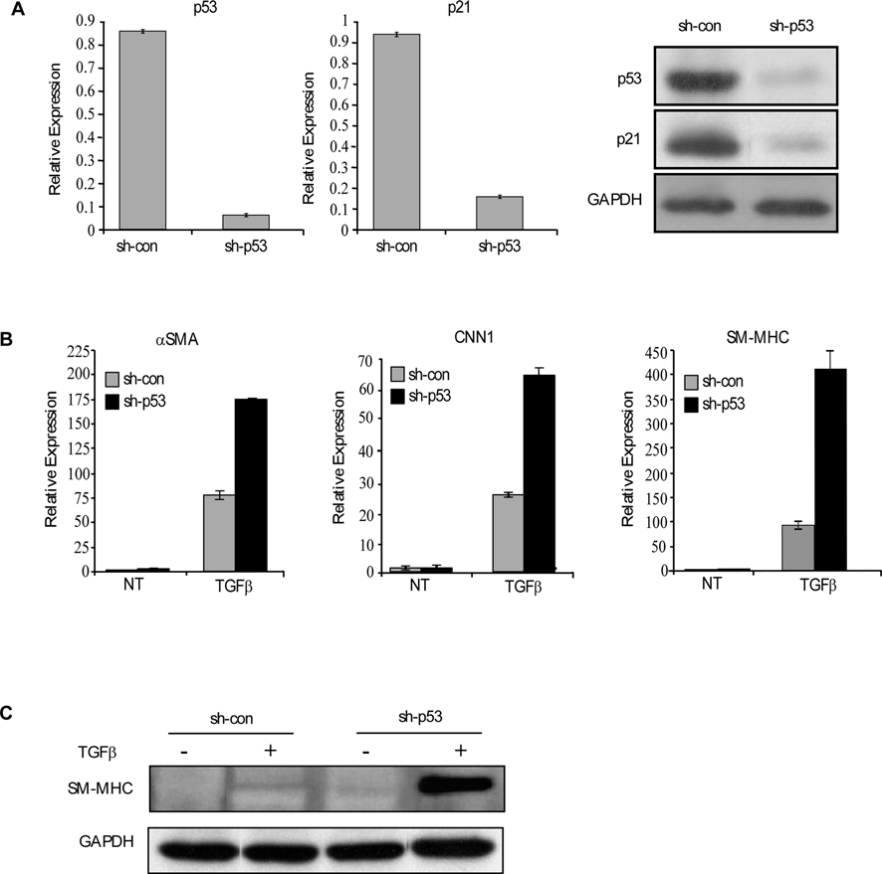
p53 negatively regulates myofibroblast/smooth muscle differentiation program. (A) h-TERT immortalized, early passaged WI-38 cells were infected with retroviruses encoding for either p53 directed sh-RNA (sh-p53) or control sh-RNA (sh-con). Relative expression levels of p53 (left panel) and p21 (middle panel) were determined by QRT-PCR, or by Western blot analysis (right panel). GAPDH serves as a loading control. (B–C) WI-38-sh-p53 and WI-38-sh-con cells were grown in culture for 48 hours (non treated, NT); serum starved for 24 hours, and then treated with a fresh serum-free medium with 1 ng/ml TGFβ for 24 h. Relative expression of αSMA, CNN1 and SM-MHC was determined by QRT-PCR (B). Equal amounts of total protein were subjected to Western blot analysis for SM-MHC. GAPDH serves as loading control (C). The results of QRT-PCR are presented as a range of two duplicate runs (N = 2) after normalization to GAPDH control.

p53 KO MEFs exhibited higher levels of smooth muscle differentiation markers (Fig 1E). However, we were not able to induce myofibroblast/smooth muscle differentiation program in MEFs. Thus, in order to study the role of p53 during smooth muscle differentiation, we used the hTERT immortalized human embryonic lung fibroblasts, WI-38, which can undergo myofibroblasts/smooth muscle differentiation, expressing smooth muscle proteins [45]. The specific role of p53 in myofibroblast differentiation is still unclear. In order to evaluate its role along this differentiation pathway, we knocked-down p53 expression in early passaged hTERT-immortalized WI-38 cells [46] by infecting them with a retrovirus encoding for human p53-directed sh-RNA. Introduction of sh-p53 resulted in a significant decrease in basal p53 protein levels as compared to cells infected with control shRNA (Fig 5A). As expected, p53 knock-down resulted in down-regulation of its transcriptional target, p21/waf1, both at the mRNA and protein levels (Fig 5A). In order to characterize the role of p53 in myofibroblast differentiation, WI-38 sh-p53 cells and their controls were treated with transforming growth factor β (TGFβ), an inducer of myofibroblast differentiation, and analyzed for the induction of several differentiation markers. In agreement with our previous study [45], we found that TGFβ treatment induced myofibroblast differentiation. Importantly, the induction of myofibroblast differentiation markers such as αSMA, CNN1 and smooth muscle-myosin heavy chain (SM-MHC), following 24 hr of TGFβ treatment, was facilitated in the sh-p53 expressing cells compared with their controls (Fig 5B). Western blot analysis revealed a dramatic upregulation of SM-MHC protein levels in WI-38 sh-p53 cells (Fig 5C). Collectively, these results imply that basal p53 activity negatively regulates TGFβ-induced myofibroblast differentiation of human fibroblasts.

To further substantiate the inhibitory role of p53 in myofibroblast differentiation, we used an additional method of p53 inactivation, namely, a dominant negative p53 peptide GSE56 [47]. Following TGFβ treatment WI-38 cells stably expressing GSE56 also exhibit augmented induction of myofibroblast differentiation markers compared with WI-38 control cells. This was evident by QRT-PCR analysis (Sup. Fig. S2).

**Figure 6:**
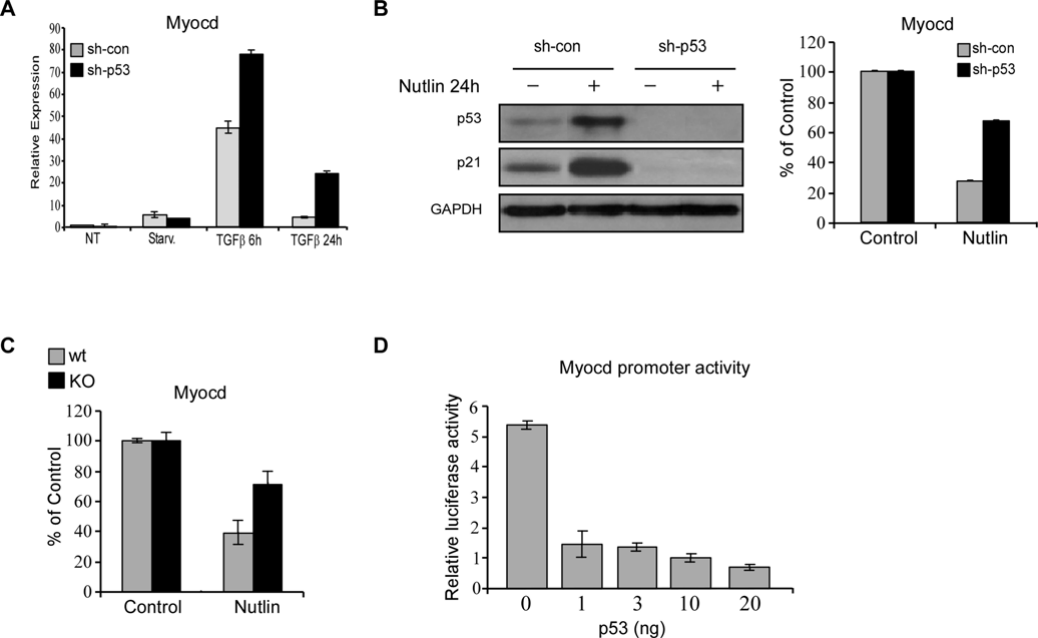
p53 downregulates Myocd expression. (A) h-TERT immortalized, early passaged WI-38-sh-p53 and control cells were grown in culture for 48 hours (non treated, NT); serum starved for 24 hours (Starv.), and then treated with a fresh serum-free medium with 1 ng/ml TGFβ for the indicated time periods. Relative expression of Myocd was determined by QRT-PCR. The results of QRT-PCR are presented as a range of two duplicate runs (N = 2) after normalization to GAPDH control. (B) h-TERT immortalized, early passaged WI-38-sh-p53 and control cells were treated with Nutlin-3 at a final concentration of 25 µM for 24 hours (Nutlin) or left untreated (Control). Western blot analysis was performed for p53 and p21.GAPDH serves as a loading control (left panel). Relative expression of Myocd was determined by QRT-PCR. Normalized expression levels in control samples were set to 100% (right panel). The results of QRT-PCR are presented as a range of two duplicate runs (N = 2) after normalization to GAPDH control. (C) p53 wt and p53 KO MEFs were grown in culture for 48 hours, then treated with Nutlin-3 at a final concentration of 25 µM for 24 hours (Nutlin) or left untreated (Control). Relative expression of Myocd was determined by QRT-PCR. Normalized expression levels in control samples were set to 100%. The results of QRT-PCR are presented as a mean±SD of two duplicate runs after normalization to HPRT control. (D) Saos-2 cells were transiently co-transfected with 300 ng Myocd promoter-luciferase reporter and the indicated amounts of wild type p53 expression plasmid. Reporter activity was determined 48 hours later. Transfection efficiency was normalized on the basis of the activity of the co-transfected renilla luciferase.

In our previous studies we demonstrated that Myocd, known as an important transcriptional regulator of smooth and cardiac muscle development, plays a key role in myofibroblast differentiation of WI-38 cells [45], [48]. As shown in Figure 6A, treatment of hTERT-immortalized WI-38 cells with TGFβ resulted in a robust induction of Myocd mRNA, reaching a peak at 6 hr of treatment. Notably, the induced levels of Myocd mRNA were significantly higher in the sh-p53 expressing cells compared to their controls. As an additional approach to test the effect of p53 on Myocd mRNA expression we used Nutlin-3. Treatment of cells with this drug resulted in accumulation of p53 and activation of its downstream target, p21 as well as in a p53-dependent downregulation of Myocd mRNA (Fig 6B). Since p53 KO MEFs express higher levels of Myocd than wt MEFs (Fig 1E), we analyzed whether induction of p53 in MEFs, following Nutlin treatment, will result in repression of Myocd also in this cell type. As evidenced in Figure 6C, this treatment resulted in ∼2.5-fold repression of Myocd mRNA in p53-proficient cells, whereas only about 30% reduction was observed in p53 KO MEFs. Thus, similarly to the situation in human embryonic fibroblasts, p53 represses mRNA levels of Myocd in MEFs.

In order to test whether p53-mediated downregulation of Myocd mRNA results from transcriptional repression of its promoter by p53, a 1.0-kb DNA fragment upstream of the transcription start site of human Myocd gene was fused to luciferase reporter gene. Reporter assays were performed in p53-null Saos-2 cells that express high endogenous levels of Myocd. As shown in Figure 6D, co-transfection of Myocd promoter reporter construct with increasing doses of wt-p53 resulted in a dose-dependent repression of the reporter activity.

Taken together, our results demonstrate that p53 downregulates Myocd both in human and murine fibroblasts. This effect is likely mediated through repression of the Myocd promoter.

### p53 positively regulates both myogenic and osteogenic differentiation of skeletal muscle committed cells

**Figure 7:**
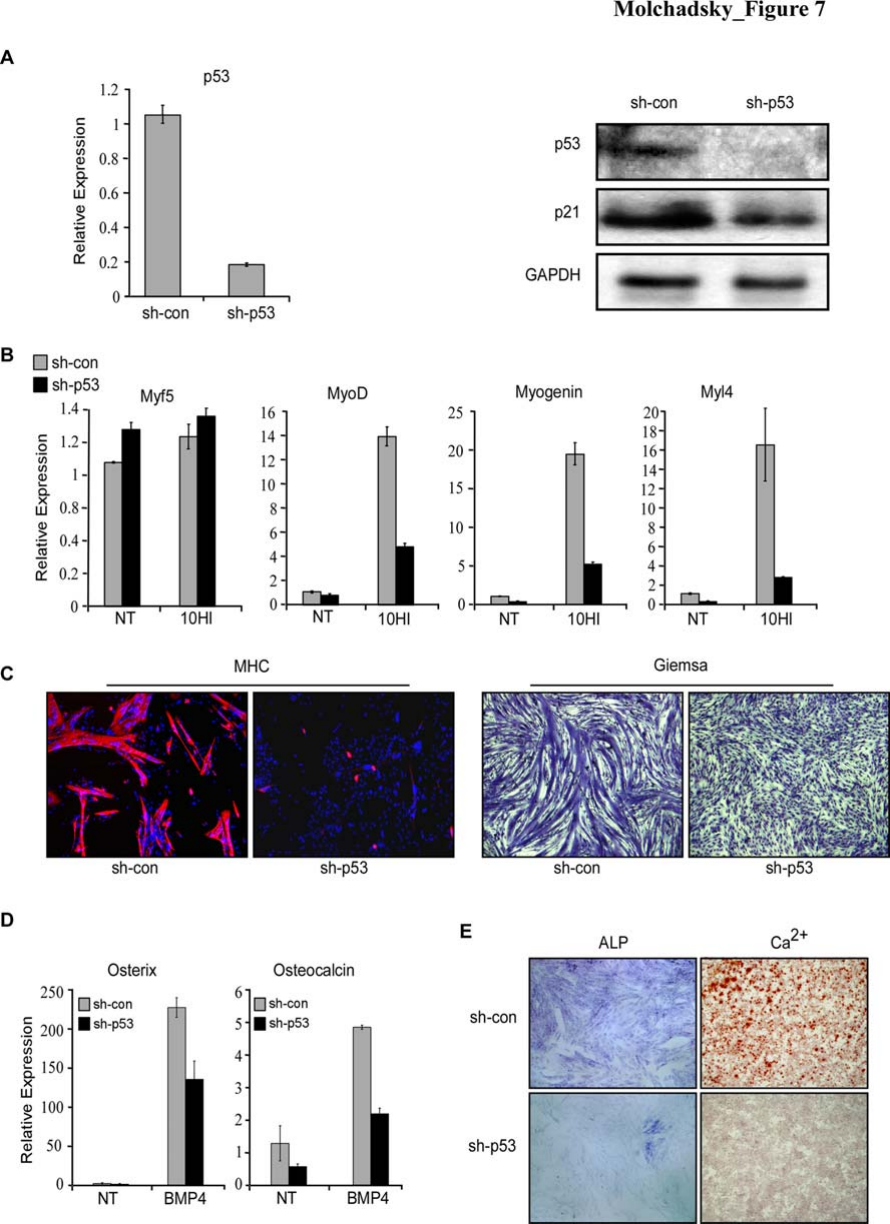
p53 positively regulates both myogenic and osteogenic differentiation of skeletal muscle cells. (A) C2 cells were infected with retroviruses encoding for either p53 directed sh-RNA (sh-p53) or control sh-RNA (sh-con). The relative expression level of p53 was determined by QRT-PCR (left panel). Western blot analysis was performed for p53 and p21. GAPDH serves as a loading control (right panel). (B–C) C2-sh-p53 and C2-sh-con cells were grown in culture for 48 hours and then subjected to induction of myogenic differentiation by treatment with medium containing 10% horse serum and 100 ng/ml insulin (10HI), or with control medium (non treated, NT) for 48 hours. Relative expression levels of Myf5, MyoD, Myogenin, and Myl4 were determined by QRT-PCR analysis (B).Myogenic differentiation was assessed using Giemsa stain and immunostaining for MHC (C). (D) C2-sh-p53 and C2-sh-con cells were grown in culture for 48 h, then medium was changed to condition medium from 293T cells overexpressing BMP4 protein or to condition medium from control cells (non treated, NT). Total RNA was isolated 48 hours later. Relative expression levels for osterix and osteocalcin were determined by QRT-PCR. The results of QRT-PCR are presented as a range of two duplicate runs (N = 2) after normalization to HPRT control. (E) C2-sh-p53 and C2-sh-con cells were grown in culture for 48 h, then medium was changed to condition medium from 293T cells overexpressing BMP4 protein. Osteogenic differentiation was assessed by measuring ALP activity and Alizarin Red staining for Ca^2+^ precipitates.

As demonstrated in Fig 1D, p53 KO MEFs express extreme levels of the skeletal muscle transcription factors, Pax3, Pax7 and Myf5, which represent early stages of skeletal muscle differentiation. Since the levels of late differentiation markers of skeletal myogenesis (e.g. myogenin and Myosin heavy chain) were not elevated even upon induction of MEFs towards the skeletal muscle lineage using horse serum and insulin, we adopted the skeletal muscle committed cell-line C2 [49] as the most suitable *in vitro* system to study the role of p53 during myogenesis. Most of previously published data suggest a positive role of p53 during skeletal muscle differentiation [33], [34], [35]. It was also suggested that the loss of p53 may be compensated by p63 and p73, the other p53 family members, and a dramatic inhibition of skeletal myogenesis is achieved when all 3 members are inactivated [33]. We first knocked-down p53 in C2 cells, by sh-RNA and verified the lack of its expression (Fig 7A). Comparative analysis of the various skeletal muscle differentiation markers between C2-sh-p53 and C2-sh-control cells revealed no differences in the levels of early markers. However, when the cells were induced to differentiate by media containing horse serum and insulin, markers that are expressed at later stages were markedly reduced in C2-sh-p53 cells compared to C2-sh-control cells (Fig 7B). These changes were in agreement with a dramatic decrease in the differentiation capacity of C2-sh-p53 compared to C2-sh-control cells, as reflected by the formation of multinucleated fibers, and the expression of myosin heavy chain in the fibers (Fig 7C).

BMP4/2 induces re-programming of myogenic cells to osteoblasts. It was previously shown that inhibition of all three p53 family members can inhibit this osteogenic differentiation of myogenic cells [33]. Due to the results demonstrating an inhibitory role of p53 in osteogenic differentiation of mesenchymal stem cells (Figs. 2, 3), we decided to analyze the effect of p53 deficiency on osteogenic differentiation of C2 cells. Surprisingly, in contrast to the negative regulatory role of p53 during osteogenic differentiation of MEFs and MBA-15 cells, p53 was found essential for both expression of osteogenic markers, and osteogenic differentiation of C2 cells (Fig 7D,E). Thus, in contrast to the inhibitory role of p53 in mesenchymal stem cells differentiation, p53 plays a positive regulatory role during the differentiation of muscle committed cells, both towards myogenesis and osteogenesis. Thereby, it can either induce or inhibit the expression of identical genes, depending on the specific cellular fate.

## Discussion

Cell differentiation represents a heterogeneous process which seems to involve various gene networks, some of which are unique to an individual differentiation program and others can be shared. By adapting a comparative study of several differentiation programs we found that p53 can play opposing roles during these programs, in a cell fate dependent manner.

Enhanced proliferation and a differentiation block are considered to be steps preceding cell transformation and tumor formation. Consequently, this agrees with the notion that p53, a tumor suppressor gene, will facilitate differentiation which is associated with cell growth arrest. Therefore, the recent data demonstrating an inhibitory role of p53 during osteogenesis of several *in vitro* and *in vivo* models warrant further assessment regarding p53 activity during normal physiological processes of cell growth and differentiation. Here we demonstrate that p53 inhibits not only osteogenesis as already shown before in other experimental systems, but also serve as an inhibitor of adipogenesis and myofibroblast differentiation of both human and mouse fibroblasts, and of mouse bone marrow stromal cells. In contrast to this broad inhibitory activity, p53 is required for differentiation of skeletal muscle cells towards mature myofibers or osteoblasts.

The involvement of p53 in osteogenesis was recently reported by several groups. Two independent studies have addressed the role of p53 in mouse differentiation models and have established that p53 functions as a negative regulator of osteoblast differentiation *in-vivo* and *in-vitro* (reviewed in [24]. Both groups show that p53 suppresses osteoblast differentiation by repressing the expression of key osteogenic transcription factors, Runx2 or osterix. Furthermore, an additional *in-vitro* study provided evidence that the absence of p53 promotes osteogenesis in mesenchymal stem cells [32]. In agreement with these findings we show, that knockdown of p53 in MEFs results in elevated expression of osterix and Runx2, as well as in higher basal and BMP4 induced ALP activity. Moreover, we present evidence that downregulation of p53 in the multipotent bone-marrow stromal cells, MBA-15, results in significant induction of their ability to undergo terminal osteogenic differentiation and form Ca^2+^ precipitates. Thus, p53 negatively regulates osteogenic differention in MEFs and bone-marrow stromal cells. While MEFs represent an early stage, reflecting the process of embryonic development, bone-marrow stromal cells are adult progenitor cells, which maintain proper bone differentiation and homeostasis.

In addition to the inhibitory role of p53 during osteogenesis, using the same cell types (primary MEFs and MBA-15) we provide compelling evidence that p53 inhibits adipogenesis *in vitro*. Our findings that p53 represses expression of the key adipogenic transcription factors PPARγ and CEBPα might represent a mechanistic basis for this effect. Although PPARγ plays a central role, as both necessary and sufficient factor during adipogenesis, there are more than one hundred other transcription factors that are expressed in adipocytes [42]. Therefore, the fact that the PPARγ antagonist, GW9662, completely blocked the accelerated adipogenic differentiation of sh-p53 MEFs (Fig. 4G), suggests that p53 might exert its inhibitory effect through this upstream key regulator of adipocyte differentiation.

In addition to its function as a regulator of adipocytes differentiation, PPARγ is expressed by several other tissues, and is involved in a number of pathological processes, such as inflammation and cancer [50]. It was found to be highly expressed by normal colonic mucosa, colorectal adenocarcinomas, and colon cancer cell lines [51], [52], [53]. Mice treated with a PPARγ ligand have greater number of polyps in the colon [54], and the levels of PPARγ mRNA in tumors of colorectal cancer patients were higher than those in adjacent normal colonic mucosa [55]. We observed upregulation of PPARγ mRNA in the colon of part of p53 null mice compared to their wt littermates (data not shown). Due to the incomplete penetrance of the p53 null phenotype, a larger group of mice should be tested to verify the specific effect of p53 deficiency on PPARγ expression, *in vivo*. Thus, while the lack of p53 in cells that can give rise to adipocytes leads to their terminal adipogenic differentiation, p53 deficiency that may lead to upregulation of PPARγ expression in colonic epithelial cells could result in their neoplastic transformation. This suggests a complex and/or indirect mechanism linking p53 and PPARγ.

Osteoblasts and adipocytes are derived from multipotent marrow mesenchymal cells, which express low levels of both adipogenic and osteogenic factors. Factors of one lineage repress factors of the other lineage, thereby maintaining the undifferentiated state of these cells. Commitment of marrow mesenchymal cells towards one of these lineages occurs when this balance is tipped leading to activation of lineage-specific transcription factors that promotes one cell fate, while repressing the other [42]. Thus, through its regulation on key osteogenic and adipogenic transcription factors, p53 may function to maintain this delicate balance, and the undifferentiated state of these stem cells, while p53 deficiency may unleash the controlled regulation of the multipotential stem state of these cells, enabling their enhanced differentiation towards both directions.

Several lines of evidence support the predicted inhibiting role of p53 in adipogenesis also *in vivo*. The physiologic role of p53 in fat metabolism is supported by the fact that p53 and its target genes are highly induced in adipocytes of the genetically obese, ob/ob mice in a fed state, leading to negative regulation of several lipogenic genes, while disruption of p53 in these mice partially restored their expression. Thus, the activation of p53 might constitute a negative feedback loop against excess fat accumulation in adipocytes [56]. In addition, mice over-expressing an active p53, exhibit reduced mean thickness of subcutaneous adipose layer [57].

Most of the fat that is stored in adipocytes serves mainly for energy production, since it is oxidized for the generation of ATP to drive metabolic processes. Thus, our data suggest that p53 may control the major metabolic energy source, by regulating the accumulation of fat storage. The role of p53 in energy metabolism was recently demonstrated [9]. The fact that p53 deficiency shifts the balance towards glycolysis rather than oxidative phosphorylation [58] means that more acetyl-CoA molecules serve as precursors for fatty acid synthesis, instead of being oxidized. Thus, p53 deficiency not only results in upregulation of key adipogenic transcription factors, but may also affect the increased production of fatty acids that are stored in fat droplets of adipocytes. It will be interesting to analyze fatty acids metabolism, *in vivo*, in p53 KO versus wt mice that were fed with a high fat diet.

The differentiation of smooth muscle is a readily reversible process in which myofibroblasts represent an intermediate stage in the phenotypic spectrum that exists between fibroblasts and smooth-muscle cells. Our results demonstrate that p53 plays an inhibitory role in this differentiation pathway. Accordingly, inactivation of p53 in cancer associated fibroblasts is predicted to contribute to their myofibroblast phenotype. Such stromal myofibroblasts, present in invasive human breast carcinomas were demonstrated to promote tumor growth and angiogenesis through elevated SDF-1/CXCL12 secretion [39]. In addition, high-grade sarcomas that form in mice following conditional mutation of K-Ras and p53 expressed markers of myofibroblastic differentiation [59]. Although the authors did not observe differences in immunohistochemical staining according to the different p53 genotypes, it would be interesting to assess the influence of p53 status on the extent of myofibroblast markers expression in this model using more quantitative methods such as RT-PCR or Western blotting..

In the case of skeletal muscle differentiation, despite the fact that we found significantly higher levels of the early markers of myogenic differentiation, Pax3, Pax7 and Myf5, in p53 KO MEFs, we failed to detect terminal differentiation towards mature skeletal muscles upon appropriate stimuli (data not shown). We also found that p53 inactivation attenuates expression of terminal markers of muscle differentiation in C2 myoblasts following differentiation induction. These results are in agreement with those of Cam and coworkers, who demonstrated that p53 plays a positive role at late stages of myogenic differentiation through transactivation of the Rb gene [33]. It was previously demonstrated that inhibition of all the p53 family members (p53, p63, and p73) inhibit skeletal myoblasts reprogramming to osteoblasts [33]. Here we demonstrate for the first time that p53 deficiency by itself is sufficient for this inhibition (Fig 7D,E). While osterix expression was upregulated in sh-p53 MEFs and MBA-15 cells, p53 deficiency led to inhibition of osterix expression in skeletal myoblasts. Thus, p53 may exert opposing effects along the same differentiation program occurring within different cell types.

Our data demonstrating opposing effects of p53 in different cell types may hold several possible explanations. One explanation could be that the function of p53 is dependent on distinct stages of mesencymal/mesodermal differentiation. We demonstrate that p53 suppresses mRNA levels of transcription factors, important at the early stages of differentiation like Myf5 and Pax3. We also confirm the results of others showing that p53 leads to repression of the osteogenic transcriptional regulators osterix and Runx2 [30], [31]. In contrast, p53 has differentiation-promoting role at late stages of several differentiation programs. For example, the early and intermediate osteogenic markers, Runx2 and osteopontin, were upregulated in p53−/− mesenchymal stem cells (MSCs) compared with wt cells during osteogenesis. However, the terminal osteogenic marker gene osteocalcin was lower in p53−/− MSCs, indicating impaired terminal differentiation [32]. Besides the transcriptional repression of transcription factors that function at early phases of differentiation programs, an additional possible explanation for the observed dichotomy of p53 function at different stages of differentiation relates to its known cell cycle inhibitory effect. Whereas early stages of differentiation are often executed concomitantly with proliferation, cell cycle arrest is associated with terminal differentiation in several mesenchymal differentiation programs. Absence of p53 facilitates cycling of MSCs but blocks subsequent terminal differentiation leading to accumulation of early and intermediate progenitors. This is reflected in the differentiation of skeletal myoblasts, where p53 may inhibit the expression of early transcription factors, but is required for cell cycle exit and terminal differentiation of these cells ([33] Fig. 1D, Fig. 7). The relationship between cell cycle inhibitory effect of p53 and its regulatory effects on the expression of master early differentiation transcription factors remains to be determined. It should be born in mind that differentiation and cell cycling are interconnected pathways, and thus it is expected that p53, shown to be involved in both, is a key factor in coordinating these pathways.

**Figure 8:**
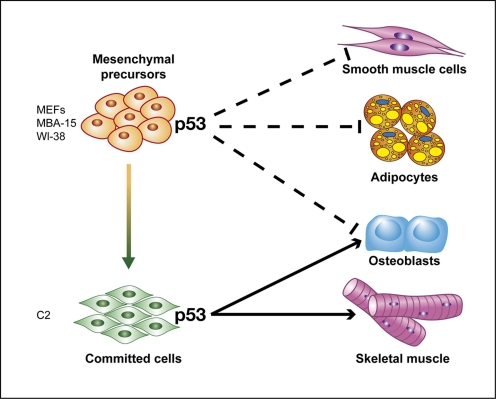
A model depicting p53 as a regulator of mesenchymal cell differentiation. p53 regulates differentiation of mesenchymal cells in a dichotomic fashion. In case of differentiation of mesenchymal precursors (MEFs, WI-38 and MBA-15), p53 attenuates myofibroblast/smooth muscle, adipogenic and osteogenic cell differentiation. In contrast, in case of more committed cells (C2), it facilitates their differentiation towards both the myogenic and osteogenic lineages.

This study suggests that p53 acts as a general regulator in cell differentiation. While in some differentiation programs p53 facilitates the process, in others it exerts suppression. In agreement with previously published data we found that p53 accelerated differentiation of skeletal muscle cells. Interestingly, we observed that p53 attenuated adipogenic cell differentiation, as well as myofibroblast/smooth muscle differentiation. In the case of osteogenesis, p53 plays a dichotomic function which is cell fate dependent. We observed that while p53 attenuates osteogenic differentiation of mesenchymal precursors (in agreement with previous reports [30], [31], [32]), it facilitates osteogenic differentiation of myogenic committed cells (Fig 8). This suggests that p53 plays a complex role in regulation of cell differentiation at large. Thus, p53 can be considered as a “guardian of differentiation” maintaining the fine balance between mutual key regulatory events during mesenchymal cell differentiation.

## Methods

### Experimental Procedures

### Cell culture and differentiation induction

Primary mouse embryonic fibroblasts (MEFs) were derived from p53 wt and p53 KO sibling embryos, and maintained with DMEM supplemented with 10% fetal calf serum (FCS) and antibiotics. The amphotropic and ecotropic Phoenix retrovirus-producing cells were from the American Type Culture Collection (Rockville, MD). The immortalized primary human embryonic lung fibroblasts (WI-38) were generated as previously described [60], and cultured in MEM supplemented with 10% FCS, 1 mM sodium pyruvate, 2 mM L-glutamine, and antibiotics. The marrow stromal cells MBA-15 [40], and the 293T cells expressing BMP-4 were maintained in DMEM supplemented with 10% FCS and antibiotics. C2 cells [549] were cultured in DMEM supplemented with 20% FCS and antibiotics.

For induction to myofibroblasts, 6×10^5^ WI-38 cells were seeded in 10 cm plates 2 days before starvation initiation. When starvation is indicated, cells were washed twice with PBS and changed to serum-free medium for 24 hr. Cells were exposed to 1 ng/ml TGFβ-1 (R&D Systems, Abingdon, UK) for the indicated time periods. For osteogenesis, MEFs were grown for a few days until subconfluence, then medium was changed to conditioned medium from 293T cells that secrete BMP-4 for the indicated time points. Alkaline Phospatase activity was detected as described [61].

For myogenesis, subconfluent C2 cells were cultured in 10HI medium (DMEM containing 10% horse serum and 0.04 units/ml insulin) for the indicated time periods. Cells were either stained with Giemsa, or fixed with 4% paraformaldehyde for 10 min, and immunostainned with anti-Myosin Heavy Chain (Clone MF-20, Hybridoma bank), as described [62]. For adipogenesis, cells were plated at a high density to reach confluence. The next day the medium was changed either to a fresh control medium or to adipogenic medium containing 10 µg/ml insulin, 10^−6^M dexamethasone, 0.5 mM 3-Isobutyl-1-methylxanthine (all from Sigma, IL). The cells were maintained for three weeks with medium replacement twice a week. Adipogenesis was detected by Oil red O staining. The PPARγ antagonist, GW9662 (Alexis Biochemicals), was added to the adipogenic differentiation medium at a concentration of 0.5 µM. Cells were fixed and stained with Oil red O after 14d. For Osteogenesis, MBA-15 cells were plated at a high density so to reach confluence. The medium was changed the next day either to a fresh control or to osteogenic medium, containing 50 µg/ml L-ascorbic acid-2 phosphate, 10 mM glycerol 2-phospate disodium salt and 10^−8^M dexamethasone (all from Sigma, IL). Osteogenic differentiation was detected by Alizarin Red staining as described [63]. For Nutlin-3 treatment, subconfluent cell cultures were treated with Nutlin-3 (Alexis Corporation, San Diego, CA) at a final concentration of 25 µM for 24 hours. Stock solution was prepared as 10 mM in DMSO.

### Retroviral Constructs and infections

pBabe-hTERT-puro was kindly provided by Dr. JW. Shay (University of Texas Southwestern Medical Center, Dallas, Texas, USA). For human p53 knockdown, the p53 short hairpin RNA (shRNA) vector, pWZL-shp53-blast and its mouse NOXA shRNA control vector, pWZL-shNOXA-blast were obtained by subcloning the p53 and NOXA fragments from pBabe-shp53- puro and pBabe-shNOXA-puro, correspondingly, which were kindly provided by Dr. D. Ginsburg (Bar-Ilan University, Ramat Gan, Israel). PLXSN-GSE56-neo was obtained from subcloning GSE56 *BamH1*fragment from pBabe-GSE56-puro which was kindly provided by Dr.AW Gudkov (Roswell Park Cancer Institute, Buffalo, NY, USA) into pLXSN.

For mouse p53 knockdown, the p53 short hairpin RNA (shRNA) vector and its human Rb shRNA control vector were kindly provided by Dr. SW. Lowe (Cold Spring Harbor Laboratory, Cold Spring Harbor, NY, USA).

Retroviral infection procedures were done using Phoenix producing cells as previously described [61].

### Quantative Real Time PCR

Total RNA was isolated using the RNAeasy kit (Qiagen) according to the manufacturer's protocol. A 2 µg aliquot of the total RNA was reverse transcribed using MMLV RT (Promega) and random hexamer primers. QRT-PCR was performed using SYBR Green PCR Master Mix (Applied Biosystems) on ABI 7000 instrument (Applied Biosystems). The values for the specific genes were normalized either to GAPDH or to HPRT housekeeping control. Specific primers were designed to the following genes: mouse HPRT: sense, GCAGTACAGCCCCAAAATGG; antisense, GGTCCTTTTCACCAGCAAGCT; mouse osterix: sense, CCTCTGCGGGACTCAACAAC; antisense, AAAGGTCAGCGTATGGCTTCTT; mouse Runx2: sense, AGGCACAGACAGAAGCTTGATG, antisense, GCGATCAGAGAACAAACTAGGTTTAGA; mouse osteocalcin: sense, GACCGCCTACAAACGCATCT, antisense, GGGCAGCACAGGTCCTAAATAGT; mouse PPARγ: TCAGGCTTCCACTATGGAGTTCA, antisense, GAAAAAACCCTTGCATCCTTCA; mouse CEBPα: sense, CGGGCAAAGCCAAGAAGTC, antisense, CGTACCCGGTACTCGTTGCT; mouse Ap2: sense, TGGAAAGTCGACCACAATAAAGAG, antisense, CACCACCAGCTTGTCACCAT, mouse AdipoQ: sense, GGATCTGACGACACCAAAAGG, antisense AGGAGAGCTTGCAACAGTAGCAT; mouse Pax3: sense, TGAGCGAGCCTCTGCACC, antisense, TCAGAGTCAATATCGGAGCCT; mouse Pax7: sense, AGCTGCTGTTGATTACCTGGC, antisense, CATACGGCGCTGTGTGGA, mouse Myf5: sense, ACAGCAGCTTTGACAGCATCTACT, antisense, GCAGCACATGCATTTGATACATC, mouse MyoD: sense, GTTCTTCACGCCCAAAAGATG, antisense, GGACAGTTGGGAAGAGTGTCATT; mouse Myogenin: sense, GTCCCAACCCAGGAGATCATT, antisense, GCAGATTGTGGGCGTCTGTAG; mouse Nkx2.5: sense, CCTCGGGCGGATAAAAAAGA, antisense, GGCTTTGTCCAGCTCCACTG; mouse Isl: sense, CGCCTTGCAAAGCGACATA, antisense, AAATTGACCAGTTGCTGAAAAGC; mouse GATA4: sense, CACCCCAATCTCGATATGTTTGAT, antisense, TGACACACTCTCTGCCTTCTGAG; mouse GATA6: sense, GGCCGTTCTTCTCGCACAT, antisense, CTACTCCAACCTGACTTTTGATTCC mouse Myocd: sense, TTACAGTTACGGCTTCAACAGAGAA, antisense, GCGGTATTAAGCCTTGGTTAGC; mouse CNN1: sense, ATGGCCAAGACAAAAGGAAACA, antisense, CTCTGCATACTTGACTCCCACATT; mouse αSMA: sense, ACATCAGGGAGTAATGGTTGGAAT, antisense, TCCCCCACATAGCTGTCTTTTT; mouse p53: sense, CACGTACTCTCCTCCCCTCAAT, antisense, AACTGCACAGGGCACGTCTT; human GAPDH: sense, AGCCTCAAGATCATCAGCAATG, antisense, CACGATACCAAAGTTGTCATGGAT; human Myocd: sense, CGAGTCTGATCCGGAGAAAGC, antisense, CTGTGAAAGAGGCCATAAAAGGTAA; human CNN1: sense, CCGTGAAGAAGATCAATGAGTCAA, antisense, CAGGTCGTTGGCCTCAAAA; human αSMA: sense, TCTAAGGCCGGCTTTGCT, antisense, CGTAGCTGTCTTTTTGTCCCATT; human SM-MHC: sense, GGATTGTGCAGACACTGAGTTTTTA, antisense, GACCGGTTACCGTGGATATGTT. The results are presented as a mean±SD of two duplicate runs from a representative experiment.

### Western blot analysis

For Western blotting, total cell extracts were prepared, 50 µg protein of each sample was fractionated by gel electrophoresis, and transferred to nitrocellulose membranes. The following primary antibodies were used: rabbit polyclonal anti-p53 (1∶1000, produced in our laboratory), rabbit polyclonal anti-p21 (1∶1000, C-19; Santa Cruz Biotechnology, Santa Cruz, CA), mouse monoclonal anti α-SMA (1∶1000, Sigma, IL), mouse monoclonal anti SM-MHC (1∶500, Sigma, IL), mouse monoclonal anti-GAPDH (MAB374; Chemicon International, Chandlers Ford, United Kingdom). Horseradish peroxidase anti-mouse and anti-rabbit (Sigma, IL) were used as secondary antibodies. The signal was detected by the super-signal-enhanced chemiluminescence system (Pierce).

## Supporting Information

Figure S1 p53 inhibits the adipogenic differentiation program in the MBA-15 cell line. (A and B) Confluent cultures of MBA-15-sh-p53 and control cells were subjected to induction of adipogenic differentiation by treatment with medium containing 10 µg/ml insulin, 10-6M dexamethasone, 0.5 Mm 3-Isobutyl-1-methylxanthine (Adipogenic medium) or with control medium (non treated, NT). The cells were grown for three weeks with medium replacement once in three days. Total RNA was isolated before to the induction of differentiation (od) as well as 7 and 21 days later. Relative expression of PPARγ, CEBPα, Ap2 and AdipoQ were determined by QRT-PCR analysis. The results of QRT-PCR are presented as a range of two duplicate runs (N = 2) after normalization to HPRT control. (A) Adipogenic differentiation was assessed using Oil Red O staining for lipid droplets (B).
(1.57 MB TIF)

Figure S2 Cells expressing a dominant negative p53 protein display enhanced myofibroblast/smooth muscle differentiation. h-TERT immortalized, early passaged WI-38 cells stably expressing GSE56 (GSE56) or control empty vector (Neo) were grown in culture for 48 hours (non treated, NT); serum starved for 24 hours, and then treated with a fresh serum-free medium with 1 ng/ml TGFβ for 24 h. Relative expression of αSMA, CNN1 and SM-MHC was determined by QRT-PCR. The results of QRT-PCR are presented as a range of two duplicate runs (N = 2) after normalization to GAPDH control.
(0.09 MB TIF)
