# Supplementary figures and images for: p53 Plays a Role in Mesenchymal Differentiation Programs, in a Cell Fate Dependent Manner
Source: PLoS One. 2008 Nov 12;3(11):e3707. doi: 10.1371/journal.pone.0003707 (PMC2577894; doi:10.1371/journal.pone.0003707)

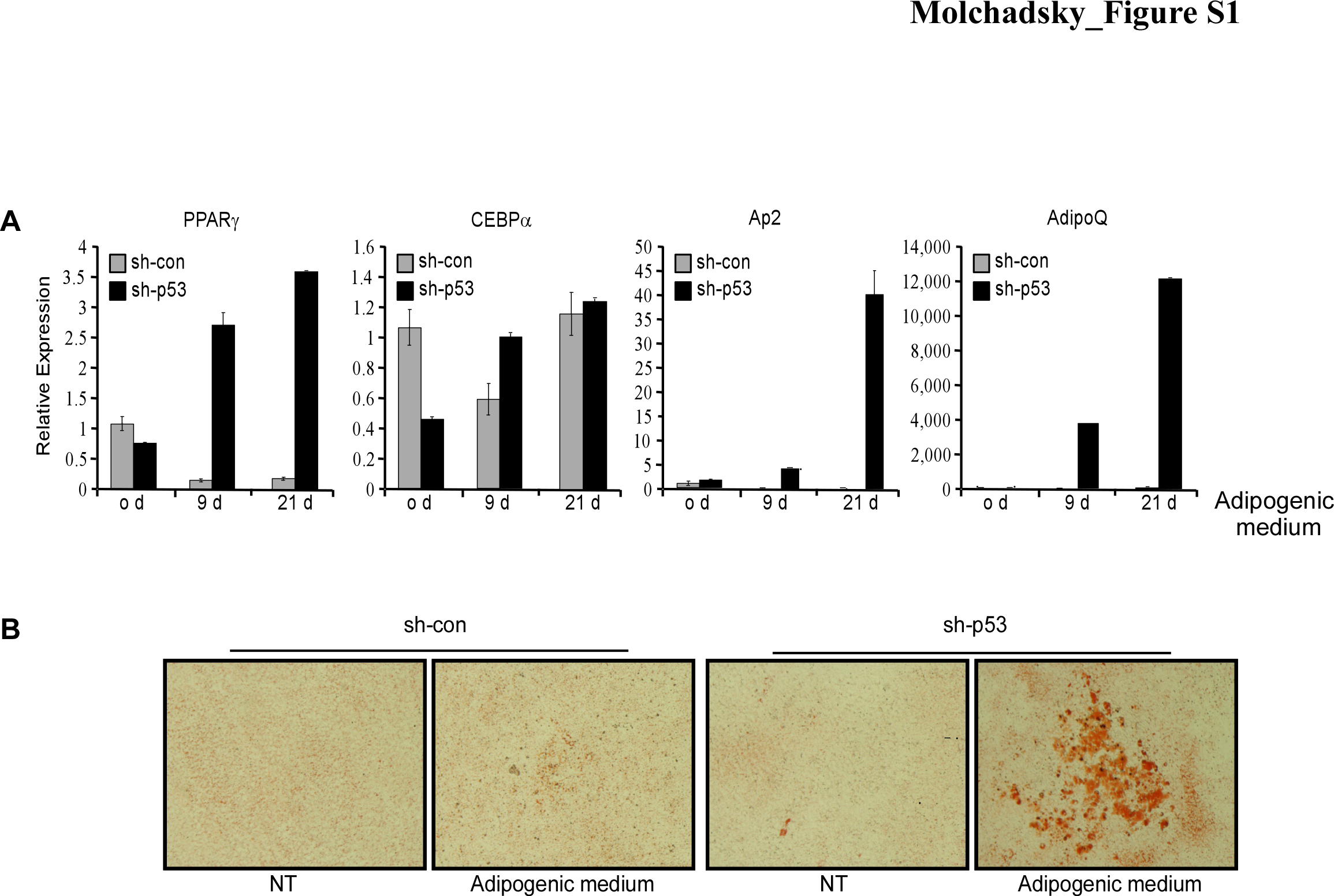

Supplement: Figure S1 — p53 inhibits the adipogenic differentiation program in the MBA-15 cell line. (A and B) Confluent cultures of MBA-15-sh-p53 and control cells were subjected to induction of adipogenic differentiation by treatment with medium containing 10 µg/ml insulin, 10-6M dexamethasone, 0.5 Mm 3-Isobutyl-1-methylxanthine (Adipogenic medium) or with control medium (non treated, NT). The cells were grown for three weeks with medium replacement once in three days. Total RNA was isolated before to the induction of differentiation (od) as well as 7 and 21 days later. Relative expression of PPARγ, CEBPα, Ap2 and AdipoQ were determined by QRT-PCR analysis. The results of QRT-PCR are presented as a range of two duplicate runs (N = 2) after normalization to HPRT control. (A) Adipogenic differentiation was assessed using Oil Red O staining for lipid droplets (B). (1.57 MB TIF) [file pone.0003707.s001.tif]

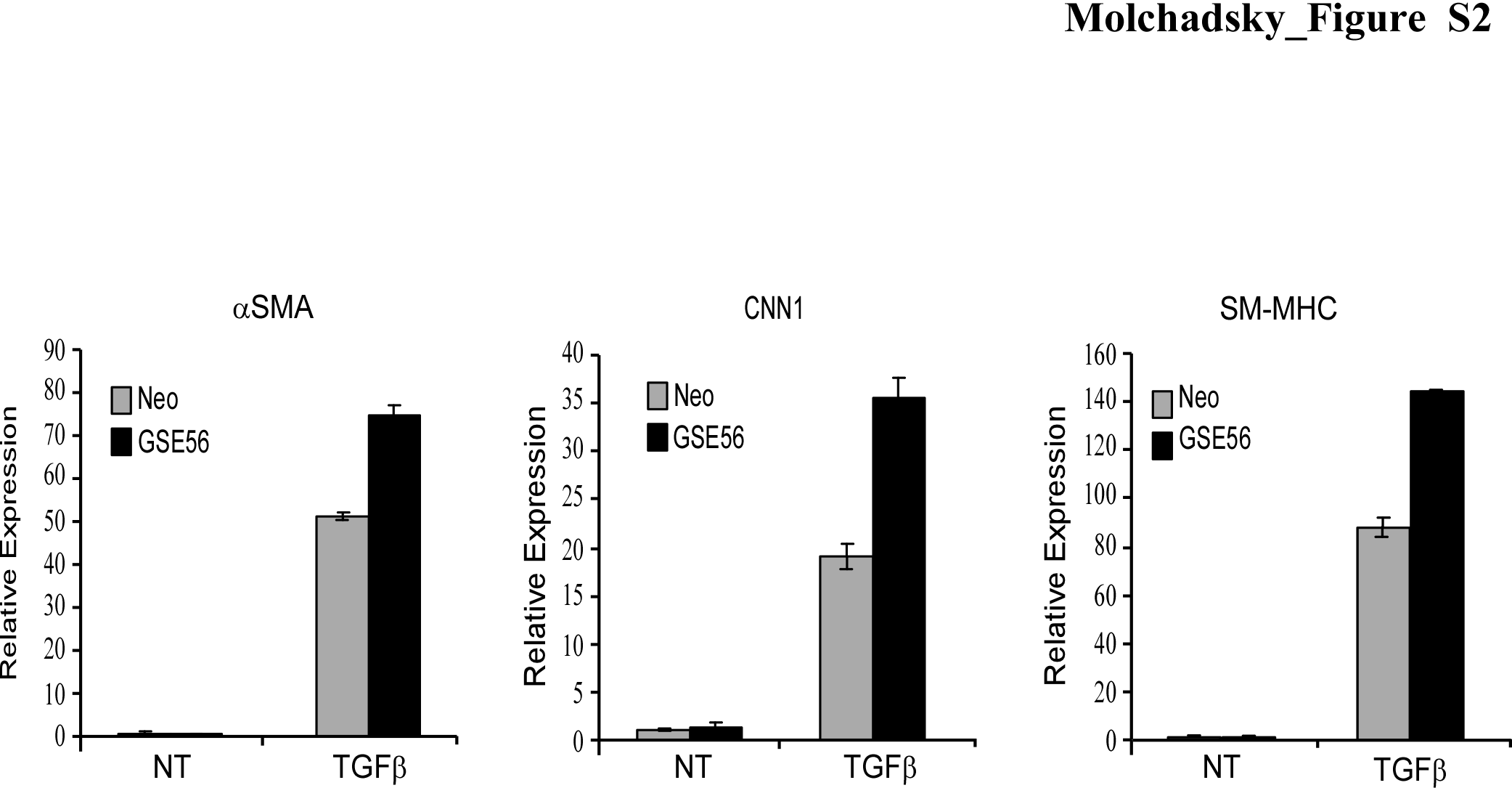

Supplement: Figure S2 — Cells expressing a dominant negative p53 protein display enhanced myofibroblast/smooth muscle differentiation. h-TERT immortalized, early passaged WI-38 cells stably expressing GSE56 (GSE56) or control empty vector (Neo) were grown in culture for 48 hours (non treated, NT); serum starved for 24 hours, and then treated with a fresh serum-free medium with 1 ng/ml TGFβ for 24 h. Relative expression of αSMA, CNN1 and SM-MHC was determined by QRT-PCR. The results of QRT-PCR are presented as a range of two duplicate runs (N = 2) after normalization to GAPDH control. (0.09 MB TIF) [file pone.0003707.s002.tif]
